# Serum Levels of Three Angiogenic Factors in Systemic Lupus Erythematosus and Their Clinical Significance

**DOI:** 10.1155/2014/627126

**Published:** 2014-01-06

**Authors:** Ling Zhou, Guoyuan Lu, Lei Shen, Linfeng Wang, Mingjun Wang

**Affiliations:** ^1^Department of Nephrology, The First Affiliated Hospital of Soochow University Suzhou, Jiangsu 215006, China; ^2^Department of Rheumatology, The First Affiliated Hospital of Soochow University Suzhou, Jiangsu 215006, China

## Abstract

Our research investigates the serum levels of three angiogenic factors in the AF family, namely, placenta growth factor (PlGF), basic fibroblast growth factor (bFGF), and vascular endothelial growth factor (VEGF), in 54 patients with SLE (SLE group) and 28 healthy controls (normal control) through ELISA measurement. And their interrelationships were also systematically analyzed. The SLE patients were then divided into active SLE group and inactive SLE group according to the SLEDAI score. The results show that serum levels of PlGF, bFGF, and VEGF in all SLE group and active SLE group were higher than those in normal controls. Serum levels of PlGF and bFGF in inactive SLE group were higher than those in normal controls. The level of PlGF was positively correlated with VEGF in SLE patients and positive correlation is also shown in bFGF with VEGF. The levels of PlGF and VEGF in SLE patients were positively correlated with both ESR and SLEDAI score. Thus a tentative conclusion can be drawn that the serum levels of the angiogenic factors, for example, PlGF, bFGF, and VEGF, may be relevant in the pathogenesis of SLE, and the concentrations of PlGF and VEGF seem to be the markers of SLE activity.

## 1. Introduction

Systemic lupus erythematosus (SLE) is a typical autoimmune disease that involves quite a few organs, with vasculitis and angiopathy as some of its typical clinical expressions [1]. The damage and activation of vascular endothelial cells are the initiation factors in the pathogenesis of SLE. Angiogenic factor (AF) is a superfamily comprising of more than 20 factors, of which the placenta growth factor (PlGF), the basic fibroblast growth factor (bFGF), and the Vascular endothelial growth factor (VEGF) are our subject of study. Previous research shows that angiogenic factors increase substantially once the damage and activation of vascular endothelial cells happen and play a significant role in vascular permeability, vascular growth, and inflammatory response. For instance, angiopoietin-2 (Angpt-2), a marker of endothelial cell activation, has been proposed as a mediator of angiogenesis, which might play an important role in the regulation of endothelial integrity and inflammation and thus is related to severity and cardiovascular disease in patients with rheumatoid arthritis [2]. And antitumour necrosis factor-*α* therapy modulates angiopoietin-2 serum levels in nondiabetic ankylosing spondylitis patients [3]. Angiogenesis may play a role in vasculitides by providing a compensatory response to ischemia and to the increased metabolic activity and may be also a further inflammatory stimulus because endothelial cells of newly-formed vessels express adhesion molecules and produce colony-stimulating factors and chemokines for leukocytes [4]. In addition, vascular endothelial growth factor (VEGF) as one of the most important proangiogenic mediators may play a role in the development of severe ischemic manifestations of giant cell arteritis [5]. Controversially, research by Rodríguez-Rodríguez et al. suggests that VEGFA polymorphisms do not seem to exert a significant influence on the risk of cardiovascular disease in patients with rheumatoid arthritis [6]. Whilst well investigated in the tumor research, the role of angiogenic factors in systemic lupus erythematosus has far been from fully understood [7]. Our clinical research aims at studying the angiogenic factors, in particular, the PlGF, bFGF, and VEGF—their expressions in the SLE patients, their interrelationships, and their correlations with other clinical indicators, by which investigating the role of AF in the pathogenesis of SLE. 

## 2. Materials and Methods

### 2.1. Participants

We identified 54 SLE in-patients within the Department of Nephrology, the Department of Rheumatology, and the Department of Dermatology in the First Affiliated Hospital of Soochow University during January 2010 and November 2010, among which 4 are males and 50 are females with mean age of  36.81 ± 12.52 years. All patients satisfied at least four items of the established American Rheumatism Association diagnostic criteria (1982) for the classification of SLE, and those patients with primary vasculitis, cerebrovascular accident, primary renal disease, tumor, and any recent infections were excluded. Among those 54 patients, 9 were newly diagnosed cases. The disease activity score of SLE was evaluated by the systemic lupus erythematosus disease activity index (SLEDAI) score, and according to it, a patient was diagnosed as active if SLEDAI score was higher than or equals to 10. Of those 54 patients in our study, 36 cases were in active SLE group and 18 cases were in inactive SLE group. In the control group there were 28 participants, all of those were healthy routine medical examinees in the First Affiliated Hospital of Soochow University during November 2010 to December 2010. Among them 6 were males and 22 were females, with mean age of 37.82 ± 12.86 years. After inquiry of medical history, medical examination, and laboratory analysis, the possibilities of other diseases or diseases of genetic inheritance were excluded. This study has been reviewed and approved by the ethics committee of the First Affiliated Hospital of Soochow University, and informed consent has been signed by all participants.

### 2.2. Lab Measurements

Venous blood of 5 mL was collected for each participant with an empty stomach, and then anticoagulated with EDTA-K2. Within 30 minutes immediately after the collection, each sample was centrifuged for 10 minutes at the speed of 3,000 r/min, so that serum samples could be extracted and then frozen and stored at −80°C for further test. The serum levels of PlGF, bFGF, and VEGF were tested through the double antibody sandwich ABC-ELISA method, with the testing kit ordered from Shanghai Westang Bio-tech Co., Ltd.. The intra and interassay coefficients of variation of all ELISA kits are less than 10%. All practical details were operated strictly in accordance with the instructions on the manual of the kit. Specimens were tested once and for all after all the collection tasks were finished. Complete blood count, blood biochemistries, humoral immunity, erythrocyte sedimentation rate (ESR), and 24-hour urine protein were routinely tested by the department of clinical laboratories of our hospital.

### 2.3. Statistical Analyses

All the quantitative data are represented as mean ± standard deviation. Independent-samples *t* test and Levene's analysis of variance are used in the comparison between groups. Pearson method is used with correlation analysis. All data are processed with statistical software SPSS 17.0.

## 3. Results

### 3.1. Clinical Data among Groups

The diastolic blood pressure (DBP) of the disease group, including the all SLE group in general and those in active SLE group and those in inactive SLE group in particular, is higher than that of the control group. Levels of hemoglobin (Hb), plasma albumin (Alb), and fasting blood glucose (FBG) in all SLE group in general and those in active SLE group and in inactive SLE group in particular are lower than those in the control group. Platelet counts (Plt) of the SLE group are lower than those of the control group. Levels of plasma triglyceride (TC) and serum creatinines (Cr-s) in the active SLE group are higher than those in the control group. Levels of blood uric acid (UA) in the inactive SLE group are lower than those of the control group. There are statistical significances in all the above differences (*P* < 0.05). There are differences on the levels of hemoglobin, plasma triglyceride, diastolic blood pressure, plasma albumin, and serum creatinine between the active SLE group and the inactive SLE group (*P* < 0.05). (Table 1).

### 3.2. Comparisons of the Levels of PlGF, bFGF, and VEGF among Groups

The levels of PlGF, bFGF, and VEGF in all SLE group and in the active SLE group are significantly higher than those in the control group (*P* < 0.01, *P* < 0.01, and *P* < 0.05). The levels of PlGF and bFGF in the inactive SLE group are significantly higher than those in the control group, and the differences have statistical significances (*P* < 0.05, *P* < 0.01). The levels of PlGF, bFGF, and VEGF in the active SLE group are higher than those in the inactive SLE group, but there is no statistical significance in the differences (*P* > 0.05). (Table 2).

### 3.3. Correlations among PlGF, bFGF, and VEGF

There are positive correlations in the level of PlGF with VEGF and in the level of bFGF with VEGF in the SLE group (*r* = 0.310, *P* < 0.05; *r* = 0.257, *P* < 0.05), while there is no correlation between the levels of PlGF and bFGF (*r* = 0.121, *P* > 0.05).

### 3.4. Correlations of PlGF, bFGF, and VEGF with Clinical Indicators

There are positive correlations in the level of PlGF with serum creatinine, erythrocyte sedimentation rate (ESR), SLEDAI score, and 24-hour urine protein (UP) and negative correlations in the level of PlGF with hemoglobin and plasma albumin. There is positive correlation between the level of bFGF and erythrocyte sedimentation rate and negative correlation between the level of bFGF and complement component C3. There are positive correlations in the level of VEGF with erythrocyte sedimentation rate, SLEDAI score, and 24-hour urine protein and negative correlation between the level of VEGF and plasma albumin. (Table 3).

## 4. Discussion

Systemic lupus erythematosus (SLE) is a rather common autoimmune disease, whose etiology or pathogenesis has not been fully understood. Deposits of the circulating immunocomplex (CIC) adhere to the inner lining of the arterial walls within the body of the patient and activate the complement pathway that generates anaphylatoxins and chemotactic factors, stimulating the white blood cells to damage the vascular endothelium, thus causing the further damages to the blood vessels and organs [8]. Under the stimulation of various pathological factors, vascular endothelial cells will release more cytokines and inflammatory mediators, causing the activation and damage of vascular endothelium, which may play a key role in the angiopathy of SLE [9].

VEGF could strongly induce the angiogenesis and play an active role in maintaining the survival of vascular endothelial cells. Recent discoveries show that there are other kinds of factors that have similar functionalities with VEGF, all of which have been generally named as the vascular growth factors, such as PlGF, bFGF, and platelet-derived growth factor (PDGF). Some recent research suggests that vascular growth factors such as VEGF participate in the pathogenesis and development of connective tissue diseases, and in SLE, any vasculitis, angiemphraxis, and vessel hypertrophy could stimulate the vascular endothelial cells to discharge or secrete vascular growth factors such as VEGF [10]. Research findings by Robak et al. [11] show that serum VEGF has a substantially high level of expression in SLE patients and is positively correlated with the ESR and SLEDAI score. These are consistent with our research findings. VEGF exerts its biological effects through binding with two high affinity tyrosine kinase receptors, namely, VEGFR-1 and VEGFR-2, of which VEGFR-1 mainly participates in the activation of angiogenesis while VEGFR-2 mediates the proliferation of epithelial cells, synthesis, and migration of DNA and the vascular permeability. Some researchers point out that the imbalance between VEGF and its two soluble receptors is one of the reasons that leads to the pathogenesis of the angiopathy of SLE [12, 13].

There are fewer investigations on the role of PlGF in the connective tissue diseases such as SLE. The amino acid sequence of PlGF is 46% homologous with VEGF. PlGF promotes human embryonic angiogenesis through binding to and activating VEGFR-1 [14] and enhances monocyte chemoattraction, vascular growth, and mobilization of bone marrow precursor cells. Research shows that besides its role on the VEGF receptors, PlGF could also participate in the angiogenesis through enabling the monocytes to secrete VEGF [15, 16]. Oura et al. [17] have observed the differences between PlGF deficient mice and wild-type mice in the cutaneous delayed-type hypersensitivity (DTH) reactions and found out that PlGF deficiency resulted in a diminished and abbreviated inflammatory response, together with a reduction of inflammatory angiogenesis and edema formation. Findings by Bottomley et al. [15] show that PlGF could strongly induce the secretion of VEGF and PPMG in patients with arthropathies. Our research finds out that the levels of PlGF in all SLE group in general and in active SLE group and in inactive SLE group in particular are all higher than those in the control group. This is in consistence with the research findings of Robak et al. [18]. Meanwhile we also find out that the level of PlGF is positively correlated with that of VEGF, ESR, and SLEDAI score in the SLE group, suggesting that PlGF is very likely to play its role in the angiopathy of SLE through enabling the secretion of VEGF and binding with it to activate VEGFR-1, and PlGF might also be relevant to the disease activities.

BFGF, as a member of the multifunctional fibroblast growth factor family, is highly active both in vivo and ex vivo in enhancing the mitosis, chemotaxis, neurotrophy, and angiogenesis. Laboratory mouse tests by Seghezzi et al. [19] find out that although there is very few expression of VEGF in resting endothelial cells, added exogenous recombinant human bFGF could stimulate the endothelial cells to synthesize VEGF and enable the cornea neovascularization, whereas VEGF antibody inhibits these. In this regard it is believed that bFGF could enhance the expression and secretion of VEGF. Previous research shows that serum bFGF has an elevated expression in connective tissue diseases such as scleroderma and dermatomyositis, whilst there are few and even controversial investigations regarding the expression of serum bFGF in SLE. Hrycek et al. [20] tested the serum level of FGF in 48 SLE patients, who were then grouped according to their status of treatment. Results showed that the level of FGF was low in patients who were newly diagnosed and only higher in those patients who had received subsequent treatment. Our research shows that serum level of FGF in all SLE group, the active SLE group, and the inactive SLE group are all significantly higher than that in the control group, and the level of bFGF is positively correlated with that of VEGF, suggesting that bFGF might, along with other factors, participate in the angiopathy of SLE by enhancing the expression and secretion of VEGF. Yet the innate mechanisms and their interrelationships of how these angiogenic factors contribute to the angiogenesis of the SLE patients still remain unclear, allowing for further investigations.

Our research findings show that the levels of PlGF, bFGF, and VEGF in active SLE group are higher than those in the inactive SLE group, but there is no statistical difference in the results. It can be explained that the angiopathy of the SLE patients in active SLE group is somewhat controlled after immunosuppression treatment and the diseases tend to ease off. But some previous findings by other researchers show that the levels of the angiogenic factors in active SLE group were significantly higher than those in the inactive SLE group [11, 20], which is inconsistent with our findings. This may be due to the fact that there are differences in the selection of individual patients and the size of the sample. This discrepancy has to be further investigated. In addition, simple correlation analysis shows that VEGF is negatively correlated with plasma albumin, and PlGF is also negatively correlated with hemoglobin and plasma albumin, suggesting that with the activity and development of the disease, the nutritional conditions of the patients gradually deteriorate, resulting in a continued increase in the serum levels of PlGF and VEGF. Our research also shows that the levels of PlGF and VEGF are positively correlated with 24-hour urine protein, and the level of PlGF is positively correlated with serum creatinines, indicating that both PlGF and VEGF might participate in the pathogenesis of lupus nephritis. Recent research by Frieri supports our view [21].

## 5. Conclusions

To sum up, it seems that PlGF, bFGF, and VEGF may be working in coordination in the pathogenesis of SLE. Meanwhile, both PlGF and VEGF could be the markers of SLE activity. Internationally, therapies of antiangiogenic factors for cancer and retinopathy have been put into clinical practice, for instance, thalidomide [22, 23] has been proved to be effective to SLE in which traditional trials have proven futile. With the research development in the expression and regulation mechanisms of autoimmune diseases, angiogenic factors are very promising in becoming new laboratory indicators and new therapies, playing their vital roles in the diagnosis, targeted therapy, and prognosis of diseases.

## Figures and Tables

**Table 1:** Clinical data among groups (mean ± standard deviation).

	Control group	All SLE group	Active SLE group	Inactive SLE group
Number of cases	28	54	36	18
Age	37.82 ± 12.86	36.81 ± 12.52	34.50 ± 11.84	41.44 ± 12.91
Gender (F/M)	22/6	50/4	34/2	16/2
SBP (mmHg)	120.11 ± 14.27	126.39 ± 27.28	130.28 ± 28.79	118.61 ± 22.81
DBP (mmHg)	72.54 ± 8.39	80.69 ± 18.867^a^	84.36 ± 19.97^b^	73.33 ± 14.25^d^
Hb (g/L)	145.89 ± 13.192	113.96 ± 20.84^a^	108.22 ± 21.71^b^	125.44 ± 13.19^cd^
Plt (109/L)	196.29 ± 39.93	167.31 ± 83.35^a^	165.36 ± 95.04	171.22 ± 55.11
TC (mmol/L)	4.89 ± 1.19	5.56 ± 2.41	5.78 ± 2.58	5.12 ± 12.03
TG (mmol/L)	1.63 ± 1.00	2.20 ± 1.43	2.42 ± 1.62^b^	1.74 ± 0.79^d^
Alb (g/L)	45.20 ± 2.42	33.42 ± 8.18^a^	31.80 ± 7.88^b^	36.68 ± 8.00^cd^
Cr-s (*µ*mol/L)	60.25 ± 13.36	86.66 ± 68.23	97.03 ± 81.57^b^	65.92 ± 11.50^d^
UA (mmol/L)	339.29 ± 91.91	314.24 ± 134.31	330.93 ± 155.32	280.85 ± 69.29^c^
FBG (mmol/L)	5.84 ± 0.97	5.10 ± 0.89^a^	5.17 ± 0.09^b^	4.95 ± 0.91^c^

Note: When compared with the control group, ^a^ *P* < 0.05, ^b^ *P* < 0.05, and ^c^ *P* < 0.05; When compared with the active SLE group, ^d^ *P* < 0.05.

**Table 2:** Serum Levels of PlGF, bFGF, and VEGF among groups (mean ± standard deviation).

Groups	PlGF (pg/mL)	bFGF (pg/mL)	VEGF (pg/mL)
Control group	41.53 ± 3.40	23.87 ± 24.53	47.29 ± 52.62
All SLE group	51.51 ± 20.75^b^	69.75 ± 88.88^b^	91.47 ± 108.67^a^
Active SLE group	54.40 ± 24.35^b^	73.49 ± 103.26^b^	100.87 ± 129.89^a^
Inactive SLE group	45.71 ± 8.20^a^	62.28 ± 50.87^b^	72.70 ± 39.05

Note: When compared with the control group, ^a^ *P* < 0.05, ^b^ *P* < 0.01.

**Table 3:** The correlations in the serum levels of PlGF, bFGF, and VEGF with each clinical data in the SLE group.

Clinical data	PlGF		bFGF		VEGF	
	*r* value	*P* value	*r* value	*P* value	*r* value	*P* value
Hb	−0.474	0.000	−0.125	0.358	−0.151	0.275
ALB	−0.311	0.022	−0.076	0.585	−0.280	0.040
Cr	−0.581	0.000	−0.038	0.787	−0.007	0.962
ESR	0.346	0.010	0.278	0.042	0.527	0.000
Complement C3	−0.210	0.081	−0.278	0.042	−0.108	0.438
SLEDAI score	0.269	0.049	0.006	0.965	0.385	0.042
24 h UP	0.345	0.034	0.010	0.879	0.457	0.013

## Abbreviation

### (in the order of their appearance in the paper)

SLE: Systemic lupus erythematosus
AF: Angiogenic factor
PlGF: Placenta growth factor
bFGF: Basic fibroblast growth factor
VEGF: Vascular endothelial growth factor
SLEDAI: Systemic lupus erythematosus disease activity index
ABC-ELISA: Avidin biotin complex enzyme-linked Immunosorbent assay
ESR: Erythrocyte sedimentation rate
DBP: Diastolic blood pressure
Hb: Hemoglobin
Alb: Plasma albumin
FBG: Fasting blood glucose
Plt: Platelet counts
TC: Plasma triglyceride
Cr-s: Serum creatinines
UA: Uric acid
UP: Urine protein
CIC: Circulating immunocomplex
PDGF: Platelet-derived growth factor
TGF: Transforming growth factor
TNF: Tumor necrosis factor
DTH: Delayed-type hypersensitivity
SBP: Systolic blood pressure
DBP: Diastolic blood pressure.
